# Individual and Organizational Factors in Coping With COVID-19 in Soldier Students

**DOI:** 10.3389/fpsyg.2022.924537

**Published:** 2022-07-05

**Authors:** Irma Talić, Alina Einhorn, Karl-Heinz Renner

**Affiliations:** Department of Psychology, University of the Bundeswehr Munich, Neubiberg, Germany

**Keywords:** military, coping, COVID-19, personality, commitment

## Abstract

The COVID-19 pandemic has posed significant burden across different industrial sectors. Generally, an increase in psychological stress experiences has been reported, while the stress and coping responses of specific, potentially burdened populations have received less attention thus far. Thus, the present study investigated relations between individual (i.e., extraversion, neuroticism, conscientiousness) and organizational (i.e., organizational commitment and study satisfaction) factors, indicators of psychological health (i.e., loneliness, life satisfaction, COVID-19-related stress), and possible mediating effects of four broad coping dimensions (active coping, avoidant coping, social support, positive cognitive restructuring) in a specific sample of soldier students who engage in a double-role being military affiliates and students of non-military subjects. To this end, we assessed data of soldier students at two measurement points (*N* = 106 at t_1_ and *N* = 63 at t_2_) shortly after the second national lockdown in Germany (20. May 2021 to 11. July 2021) during the COVID-19 pandemic. Personality traits showed expected associations with indicators of psychological health, i.e., positive relations between neuroticism and social loneliness, between extraversion and COVID-19 stress, and negative relations between neuroticism and life satisfaction. Remarkably, organizational variables showed effects above and beyond personality traits on loneliness and life satisfaction. Neither individual, nor organizational factors could predict change in psychological health over time. We found evidence for mediation effects through active coping, avoidant coping, and the use of social support, but not through positive cognitive restructuring. Findings highlight the relative importance of organizational factors besides personality traits for psychological health in a military student sample, holding important implications for designing efficient support systems in the military.

## Introduction

The COVID-19 pandemic has been an unprecedented event in most peoples’ lives. The confrontation with an unknown virus was associated with various stressors (e.g., fear of infection, hospitalization, threat of economic losses, isolation or quarantine after government-implemented containment measures and even national lockdown phases; Hale et al., 2020). Affecting individual, family, educational, occupational, and medical systems, the COVID-19 pandemic has been conceptualized as a multidimensional stressor causing substantial psychological distress (Gruber et al., 2021). Because of this stressful impact and the worldwide spreading of the virus, the COVID-19 pandemic may also be framed as a *critical world event* alluding to the concept of critical life events in stress research (Holmes and Rahe, 1967). Soon, the importance of examining the psychological impact of the COVID-19 pandemic has been highlighted, particularly with regard to understanding human behavior with the ultimate goal of coping efficiently with the virus and containing its spread as well as to tailoring effective interventions for those in need (Kazak, 2020).

Psychological health has been shown to be substantially affected by the COVID-19 pandemic resulting in a set of responses across people, including for instance depression, anxiety, panic attacks, somatization and sleep disorders (Hossain et al., 2020). However, as well established in stress and coping research before the pandemic, individual differences play a decisive role in the face of adversity (Bonanno, 2004; Seery et al., 2010; Masten, 2011; Hamby et al., 2018). There is some evidence of personality traits (e.g., Aschwanden et al., 2020; Zettler et al., 2022), as well as coping strategies (Zacher and Rudolph, 2021b) as predictors of COVID-19-related psychological health outcomes. Yet, these studies focus on samples from the general population. Among others, substantial psychological burden has been reported for college students (e.g., Wang et al., 2020), as well as for certain professional groups including health workers (Giorgi et al., 2020), police (Frenkel et al., 2021), and the military (Gordon et al., 2021), indicating the need to address specific populations and their respective crucial protective and vulnerable factors in examining psychological health outcomes following stress related to the COVID-19 pandemic.

In order to meet this need, the present study examined a population of soldier students (i.e., young officer candidates currently undergoing studies at the University of the Bundeswehr Munich) of the German Federal Armed Forces (*Bundeswehr*)—a sample that comprises both a military affiliation and non-military university studies. Taking account of the German *Bundeswehr* as “special organization” (Richter, 2017), we incorporated the examination of organizational factors above personality traits as potentially crucial predictors of psychological health in this sample. We further assessed different strategies in coping with the COVID-19 pandemic, which we tested as potential mediators between individual and organizational factors and psychological health (i.e., loneliness, life satisfaction, COVID-19 stress). In doing so, we shed some light on resilience and vulnerability in a military student sample during the COVID-19 pandemic.

## Psychological Effects of COVID-19

On 11. March 2020, the World Health Organization (WHO) declared the COVID-19 outbreak a global pandemic, massively disrupting daily life of many people ever since. Until the onset of our study in May 2021, Germany had terminated its 6-month, second national lockdown with travel prohibitions, curfews, contact bans and closure of cultural and sports facilities, as well as partial retail sector and school closures (Moradian et al., 2021). During this second lockdown, a high ongoing psychological burden (i.e., COVID-19-related fear, generalized anxiety, depressive symptoms, and psychological distress) was reported in a German sample (Moradian et al., 2021). From May 2021 on, a stepwise cautious relaxation of measures was implemented in accordance with infection rates (e.g., weakened contact bans, only regional curfews, opening of the food service sector or enabling touristic travels under strict conditions). Up until the onset of our study (i.e., 20. May), COVID-19 associated infections [deaths] reached 3,638,504 [87,135], and 32,961,750 people (about 39.29% of the German population) received at least one vaccination (Dong et al., 2020).^1^

This extraordinary situation showed an impact on various psychological health outcomes, including increased loneliness (i.e., painful subjective feelings of isolation and with this an indicator of social well-being; Ellis et al., 2020; Huxhold and Tesch-Römer, 2021; Ernst et al., 2022), decreased life satisfaction (i.e., the cognitive evaluation of one’s own subjective well-being; Zacher and Rudolph, 2021b), and increased psychological distress with anxiety and depressive symptoms (Arslan et al., 2021; for an overview see Hossain et al., 2020). While these findings indicate substantial COVID-19-related psychological distress, researchers also reported heterogeneous results (Ernst et al., 2022) or resilience (Luchetti et al., 2020), motivating research on potential vulnerability and protective factors.

## Personality and COVID-19

Generally speaking, personality consists of all outlasting individual characteristics of a human being in terms of bodily appearance, and patterns of behavior and experiences (Asendorpf, 2019). The personality traits extraversion, neuroticism, and conscientiousness are similarly related to psychological and subjective well-being and therefore considered personality predispositions for general well-being (Grant et al., 2009). Extraversion reflects an individual’s tendency for sociability, activity and is often accompanied by experiencing positive emotions (e.g., joy), while neuroticism implicates emotional instability, impulsivity, fear, and anger and can be interpreted as a general predisposition to experiencing psychological distress (Costa and McCrae, 1980, 1992). Finally, conscientiousness describes organization and diligence in task completion (Costa and McCrae, 1992). Research prior to the pandemic indicated that extraversion was positively related to subjective well-being (Guitiérrez et al., 2005; Soto, 2013; Weninger and Holder, 2013), and negatively related to depression and anxiety (Jylhä and Isometsä, 2006). People with higher values in extraversion tended to have larger social networks that offer more social support (Asendorpf and Wilpers, 1998). During the pandemic, extraverted people tended to perceive government-implemented measures as stricter (Modersitzki et al., 2021) and to engage less in social distancing (Carvalho et al., 2020). Extraversion was positively related to higher average levels of perceived stressfulness of the pandemic (Zacher and Rudolph, 2021a) and seemed to have partially lost its protective role for subjective well-being (Anglim and Horwood, 2021) and against loneliness in times of social/physical distancing (Gubler et al., 2021). Overall, extraversion was negatively related to generalized anxiety and depressive symptoms (Nikčević et al., 2021) and positively with psychological well-being (Shokrkon and Nicoladis, 2021) and life satisfaction (Modersitzki et al., 2021) during the pandemic. When considering phases of the pandemic in a longitudinal study design, extraversion was related with both increases and decreases in perceived stressfulness in accordance with external changes (Zacher and Rudolph, 2021a).

Neuroticism was found to be a factor of vulnerability for life stress and changes (Suls and Martin, 2005) and a risk factor for depression and anxiety (Roelofs et al., 2008) before the pandemic. Neuroticism consistently showed negative relations to subjective well-being and life satisfaction (e.g., Librán, 2006; Gale et al., 2013; Soto, 2013), and relationship satisfaction (Vater and Schröder-Abé, 2015). During the pandemic, negative relations between neuroticism and subjective well-being and life satisfaction were replicated (Modersitzki et al., 2021). Persons scoring higher in neuroticism further tended to display more COVID-19-related concerns (Aschwanden et al., 2020), lower psychological well-being (Shokrkon and Nicoladis, 2021), and more generalized anxiety and depressive symptoms during the pandemic (Nikčević et al., 2021).

Conscientiousness was reported to be a positive predictor of subjective well-being (Grant et al., 2009), and showed positive relations to daily life satisfaction (Smith et al., 2013) before the pandemic. During the pandemic, conscientiousness was related with greater adherence to social distancing measures (Carvalho et al., 2020) and more precautions (Aschwanden et al., 2020). Further, conscientiousness was negatively related to generalized anxiety and depression (Nikčević et al., 2021) and positively related to life satisfaction (Anglim and Horwood, 2021; Modersitzki et al., 2021) during the pandemic.

## Coping Strategies, Personality, and COVID-19

According to the Transactional Model (Lazarus and Folkman, 1984), coping is defined by thoughts and behaviors that are used with the aim of managing a person-environment transaction that is appraised as stressful (Folkman and Moskowitz, 2004). Different individual coping strategies can be clustered within the four higher-order coping strategies active coping (e.g., planning), avoidant coping (e.g., substance use), use of social support (e.g., emotional support), and positive cognitive restructuring (e.g., acceptance; Nahlen Bose et al., 2015; Baumstarck et al., 2017). The efficiency of these coping strategies for maintaining psychological health in adverse circumstances highly depends on the context, in which they are applied (Folkman and Moskowitz, 2004). In the context of the COVID-19 pandemic, Zacher and Rudolph (2021b) reported positive relations between active coping and positive cognitive restructuring with regard to life satisfaction and negative relations between planning and life satisfaction. The use of social support was related to an increase in life satisfaction (for instrumental support) in addition to higher levels of positive affect and lower levels of negative affect (for emotional support), but also higher levels of negative affect (for instrumental support; Zacher and Rudolph, 2021b), and showed low levels of well-being (Kavčič et al., 2022), supporting the notion of social support as “double-edge sword” (Carver et al., 1989; Revenson et al., 1991). Avoidant coping strategies were positively related with higher negative affect (Zacher and Rudolph, 2021b), depressive symptoms, anxiety, and stress (Agha, 2021; Minahan et al., 2021; Kavčič et al., 2022).

In turn, certain personality traits are related to the use of certain coping strategies. Before the COVID-19 pandemic, a meta-analysis showed extraversion and conscientiousness to be positively related to active coping and positive cognitive restructuring, while extraversion and neuroticism were both positively related to seeking social support (Connor-Smith and Flachsbart, 2007). Neuroticism further showed positive relations to avoidant coping, and negative relations to active coping and positive cognitive restructuring (Connor-Smith and Flachsbart, 2007). There is a lack of studies on relations of personality traits and coping strategies during the pandemic. Agbaria and Mokh (2021) report positive correlations between emotion-focused coping (that comprises avoidant coping and positive cognitive restructuring in their study) and neuroticism, and negative correlations between emotion-focused coping and both extraversion and conscientiousness, while active coping was positively related to extraversion and conscientiousness and negatively related to neuroticism.

## Soldier Students in the German *Bundeswehr* and COVID-19

The COVID-19-related studies reported as of yet mostly focus on samples from the general population, whose findings do not seem readily generalizable to other specific samples such as soldier students. For instance, at the individual level, there is evidence for personality differences in people in emergency-service professions (i.e., the “Rescue Personality”; Klee and Renner, 2013, 2016; Mitchell, 1983; Salters-Pedneault et al., 2010). At the organizational level, military organizations are distinct from other organizations in a number of features (e.g., special forms of socialization, a pronounced importance of symbols and rituals, military-specific camaraderie, the principle of order and obedience; Richter, 2017), thus contributing to a military identity (Kümmel, 2018).

The University of the Bundeswehr Munich is one of two universities of the *Bundeswehr* in Germany that are subordinated to the Federal Ministry of Defence on the one hand, but also the Higher Education Act like the regular state universities on the other hand. Students enrolled in the University of the Bundeswehr Munich are soldiers (i.e., officer candidates or lieutenants) who undergo an academic education and obtain Bachelor and Master degrees in non-military subject areas (e.g., economics, computer science, educational science) that are equivalent to degrees obtained from state universities, thus standing in contrast to military academies (e.g., United States Military Academy in West Point, New York or the Royal Military Academy Sandhurst).

The German *Bundeswehr* played a special role in fighting the pandemic. Since the onset of the pandemic in March 2020, soldiers in Germany were involved in various COVID-19-related tasks including assistance with material and logistics (e.g., providing face masks), tracing infection chains, testing for the virus in care facilities for the elderly, and operating own vaccination centers (Bundeswehr, 2022). The assistance provided by the *Bundeswehr* in the combat against the COVID-19 pandemic was primarily performed by professional military and only in some cases by soldier students of the University, that had the opportunity and were encouraged to engage in various COVID-19-related chores (e.g., providing assistance to local health offices), but were not obligated to do so. However, substantial burden during the pandemic in this sample can still be assumed due to a number of reasons. Soldier students find themselves in a double role being soldiers (i.e., officer candidates or lieutenants) and students at the same time, involving primarily study obligations along with certain military obligations. Considerable psychological burden (i.e., symptoms of depression and anxiety and even suicidal thoughts) has been reported for college students during the pandemic (Wang et al., 2020). In contrast to regular college students, soldier students undergo intensive study programs structured within trimesters that allow for a shorter study duration. They have the order to complete their studies successfully as part of their qualification as an officer, and also receive pay during their 4 years of study. Therefore, the study duties can also be described as work demands rather than regular study demands, potentially suggesting increased study burden as compared to regular student samples. Moreover, soldier students experienced massive changes in their habitual daily life during the pandemic. Usually sharing barracks on an on-site campus, they were freed from the obligation to stay at this campus. Life at campus was subjected to various regulations (e.g., strict restrictions in performing sports activities). Teaching and study obligations were exclusively performed online (with the exception of exams). At the organizational level, such changes have been shown to be associated with a higher risk for psychological health problems before the pandemic (Bamberger et al., 2012).

In addition to organizational changes, organizational commitment as well as job satisfaction are dominantly related to employee well-being and health (e.g., Panaccio and Vandenberghe, 2009; Donaldson and Ko, 2010; Faragher et al., 2013; Rodríguez-Fernández et al., 2021). Organizational commitment is a “bond or linking of the individual to the organization” (Mathieu and Zajac, 1990), whereas its subcomponent affective organizational commitment has been described as a “core essence” (Mercurio, 2015) of organizational commitment and comprises feelings of shared values, pride, affiliation and identification with organizational goals (Felfe and Scherm, 2012).^2^ Job satisfaction refers to an individual’s positive emotional responses and attitudes toward (aspects of) their job (Faragher et al., 2013). Similarly, study satisfaction refers to an individuals’ satisfaction or dissatisfaction with (components of) their studies (Westermann et al., 2018).^3^

Before the pandemic, job satisfaction and organizational commitment reported to be predictors of psychological health, showing significant positive relations to life satisfaction and negative relations to job stress in police officers (Moon and Jonson, 2012; Lambert et al., 2021), a profession related to military personnel. Similarly, job satisfaction and organizational commitment have been shown to be directly affected by work stress levels in military personnel (Dobreva-Martinova, 2002). In a meta-analysis, however, affective organizational commitment turned out to be a crucial factor for lower stress levels and absenteeism, and higher performance and work engagement (Meyer et al., 2002; see also Rivkin et al., 2018). Job satisfaction and loneliness were negatively related before the pandemic (Tabancali, 2016; Bakır and Aslan, 2017). The pandemic implicated massive changes at the work place (e.g., social distancing and loneliness, working from home, the distinction between “essential” (i.e., life-sustaining) and non-essential workers) and typically an increase in work stress (Kniffin et al., 2021). At the same time, individual (e.g., personality) and organizational (e.g., organizational culture) differences were discussed as potential moderators of psychological health outcomes in this crisis (Kniffin et al., 2021), motivating the examination of individual and organization predictors of psychological health in a sample with distinct personal and organizational features (i.e., soldier students).

## The Present Study

The present study aims to bridge the gap between research in personality, organization, and coping in a highly extraordinary and dynamic context in a specific military student sample. Personality traits have been associated with psychological stress and satisfaction with new working conditions during the pandemic in a related profession (i.e., the police; Langvik et al., 2021), yet, coping strategies were not considered. In the present study, we examined the effect of three personality traits (i.e., extraversion, neuroticism, and conscientiousness) as individual factors, and organizational commitment and study satisfaction as organizational factors, on psychological health (i.e., loneliness, life satisfaction, and COVID-19 stress) directly and indirectly through coping strategies in soldier students. In doing so, we highlight the special role of specific populations in pandemic situations, and adopt a “resilience perspective” (Chen and Bonanno, 2020)—investigating psychological health incorporating factors of both resilience and vulnerability.

While the associations between personality traits and psychological health during the COVID-19 pandemic have been investigated in some studies (e.g., Anglim and Horwood, 2021; Nikčević et al., 2021; Zacher and Rudolph, 2021a), there is considerably less research on the relation between organizational factors (i.e., organizational commitment and study/job satisfaction) and psychological health during the pandemic as well as its incremental validity over and above personality traits. To this end, we derived the first research question:

RQ1. *Cross-sectional direct analyses*: How are individual and organizational soldier student factors related to loneliness, life satisfaction, and COVID-19 stress at the same time point?

Specifically, we anticipated relations in accordance with prior research during the pandemic (for personality traits; see Carvalho et al., 2020; Anglim and Horwood, 2021; Gubler et al., 2021; Modersitzki et al., 2021; Nikčević et al., 2021; Shokrkon and Nicoladis, 2021; Zacher and Rudolph, 2021a) or before the pandemic, respectively (for organizational factors; see Dobreva-Martinova, 2002; Meyer et al., 2002; Moon and Jonson, 2012; Rivkin et al., 2018; Lambert et al., 2021). All expected relations are displayed in Table 1.

**Table 1:** Expected relations between personality traits and organizational predictors, coping dimensions, and psychological health outcomes.

**Predictors**	**Expected relation with cross-sectional psychological health outcomes**			
	**Social loneliness**	**Emotional loneliness**	**Life satisfaction**	**COVID-19 stress**
Extraversion	−	−	+	+
Neuroticism	+	+	−	+
Conscientiousness	+	+	+	−
Commitment	−	−	+	−
Study satisfaction	−	−	+	−
Active coping	−	−	+	−
Avoidant coping	+	+	−	+
Use of social support	−	−	+	0
Positive cognitive restructuring	−	−	+	−
	**Expected relation with coping dimensions**			
	**Active coping**	**Avoidant coping**	**Use of social support**	**Positive cognitive restructuring**
Extraversion	+	0	+	+
Neuroticism	−	+	+	−
Conscientiousness	+	−	0	+
Commitment	+	−	0	+
Study satisfaction	0	−	0	0

+, Expected relation is positive; −, expected relation is negative; and 0, expected relation is zero. Note that all indirect effects that suggest a (partial) mediation can be obtained by multiplying the direct effects; i.e., two positive and two negative direct effects yield a positive indirect effect, one positive and one negative direct effect yield a negative indirect effect, and if at least one direct effect is zero, the indirect effect will be zero.

The pandemic was a highly dynamic situation with various rapidly changing regulations and infection rates. Only few studies examined the predictability of change in psychological health indicators over time during the pandemic (for exceptions see Zacher and Rudolph, 2021a,b). Predictions of change hereby showed (a) little change in subjective well-being across time points that were 1 or 3 month(s) apart (Zacher and Rudolph, 2021b), and (b) predictions of change that depended on the phase of the pandemic (e.g., an association of extraversion with an *increase* in perceived stressfulness in one time frame, and an association of extraversion with a *decrease* in perceived stressfulness in another time frame; Zacher and Rudolph, 2021a). Thus, the predictability of change seems substantially complicated in such dynamic contexts. To examine the predictability of possible change in loneliness, life satisfaction, and COVID-19 stress by personality traits and organizational factors, we posed the second research question:

RQ2. *Longitudinal direct analyses*: Can individual and organizational soldier student factors predict change in loneliness, life satisfaction, and COVID-19 stress across 4 weeks?

Considering the lack of research on the predictability of change and its high context dependency, we examined RQ2 in an explorative manner and did not derive specific expectations.

Finally, to gain insight into the mechanisms of the prediction of psychological health by individual and organizational factors, we examined four broad coping dimensions as potential mediators based on theoretical considerations as described in the Transactional Model (Lazarus and Folkman, 1984) as well as relations between personality traits and coping strategies (e.g., Connor-Smith and Flachsbart, 2007), between coping strategies and psychological health (e.g., Deckx et al., 2018), and evidence for mediated personality-health associations through coping (e.g., Peng et al., 2012). We identified less studies examining the relation between organizational factors, coping, and psychological health, which we implemented in the third research question:

RQ3. *Mediation analyses*: Do coping strategies mediate the relationships between individual and organizational soldier student factors and loneliness, life satisfaction, and COVID-19 stress?

Expected relations between personality and organizational predictors and coping strategies (for personality see Connor-Smith and Flachsbart, 2007; Agbaria and Mokh, 2021; for organizational predictors see Srivastava and Tang, 2015; Portero de la Cruz et al., 2020; Rojas et al., 2022) are presented in Table 1 along with expected relations between coping strategies and psychological health outcomes (Deckx et al., 2018; Gori et al., 2020; Minahan et al., 2021; Zacher and Rudolph, 2021b; Kavčič et al., 2022). The respective expected indirect effects can be obtained by multiplying the two direct effects (i.e., two positive and two negative direct effects yield a positive indirect effect, one positive and one negative direct effect yield a negative indirect effect, and if at least one direct effect is zero, the indirect effect will be zero).

## Materials and Methods

### Procedure and Participants

The present study was administered as a longitudinal online study within two measurement waves, where all variables where assessed at both measurement points. Using the university-wide e-mail distribution system, the invitation link for the study was shared with all soldier students at the University of the Bundeswehr Munich, such that every student had the opportunity to participate irrespective of subject area or study year. The first measurement wave (t_1_) was carried out between 20. May and 8. June 2021 and the second measurement wave (t_2_) was conducted between 21. June and 11. July 2021. Study participation at t_1_ did not require participation at t_2_. If consented to participate at t_2_, data collection was administered such that there was a time gap of approximately 4 weeks between individual assessments. We assessed data from *N* = 106 soldier students at t_1_ (Sample t_1_ with *M*_age_ = 23.6 years, *SD* = 3.30; range = 19–33 years) of whom 52.8% were male. Students studied in various subject areas (e.g., aerospace engineering, psychology, sports sciences, computer science, cyber security, management and media, social sciences and public affairs, economics and management studies) at the University of the Bundeswehr Munich. We assessed data from students across all years of study with 42.3% in their 1st year of study, 22.7% in their second, 15.5% in their third, and 19.6% in their 4th year of study. At t_2_, *N* = 63 soldier students participated (sample t_2_ with *M*_age_ = 23.5 years, *SD* = 2.97; range = 19–33 years) of whom 52.4% were male. Thus, we obtained data from *N* = 63 at both measurement points (Sample t_1_ + t_2_). Since the students had the opportunity to obtain permission to leave the barracks on campus for specific times during the COVID-19 pandemic, we assessed the number of days that the study participants spent at campus during the month prior to our study (i.e., presence at campus). 17.9% of study participants reported having been completely absent, 31.1% reported having spent 1–6 days at campus, 11.3% reported having spent 7–13 days at campus, 8.5% reported having spent 14–19 days at campus, 18.9% reported to having spent 20–26 days at campus and 12.3% reported having spent more than 26 days at campus.

Students’ participation in the study was voluntary and was compensated with course credit for psychology students. Students did not receive any monetary compensation for their participation. The institutional review board of the University of the Bundeswehr Munich approved of all procedures.

### Measures

All variables were assessed at both measurement points (t_1_ and t_2_).

#### Individual Predictor: Personality

We assessed the three personality traits extraversion, neuroticism, and conscientiousness using the respective subscales from the German version (Danner et al., 2016) of the Big Five Inventory 2 (Soto and John, 2016). The three traits were assessed with 12 items each. Example items and scale reliabilities reported by Danner et al. (2016) include “*I am someone who is outgoing, sociable*” (for extraversion, *α* = .86), “*I am someone who is moody, has up and down mood swings*” (for neuroticism, *α* = .88), and “*I am someone who tends to be disorganized*” (for conscientiousness, negative indicator, *α* = .88). Participants responded on a five-point Likert scale ranging from 1 (*completely disagree*) to 5 (*agree completely*).

#### Organizational Predictors

##### Organizational Commitment

To assess the organizational commitment toward the German armed forces (i.e., the German *Bundeswehr*), we used six items of the COMMIT (Felfe and Pundt, 2012) focusing on affective organizational commitment and adapted the item wordings naming the German armed forces as organization. An example item is “*In general, I am proud to be a member of the Bundeswehr.*” Participants responded on a five-point Likert scale ranging from 1 (*does not apply*) to 5 (*completely applies*), such that higher ratings reflected higher affective organizational commitment. Felfe and Pundt (2012) reported an internal consistency of *α* = .86 for the affective organizational commitment subscale.

##### Study Satisfaction

Study satisfaction was assessed as an approximation to job satisfaction due to the mixture of study and work demands in our sample (see Section Soldier Students in the German *Bundeswehr* and COVID-19 for detailed information). Soldier students’ study satisfaction during the COVID-19 pandemic was assessed with the FB-SZ-K questionnaire (Westermann et al., 2018). We explicitly asked participants to refer to the online-teaching conditions during the pandemic when responding to all statements. The questionnaire measures three components of study satisfaction; i.e., satisfaction with study content (e.g., “*I am really happy with what I study,*” *α* = .87), satisfaction with study conditions (e.g., “*I would wish for better study conditions at the university*,” negative indicator, *α* = .71) and satisfaction with mastering study loads (e.g., “*Studies are killing me,*” negative indicator, *α* = .71) with three items each (Westermann et al., 2018). We adapted two items of the subscale satisfaction with study conditions to represent COVID-19-related adjustments at the university (i.e., “*I would wish for better study conditions at the university during the COVID-19 pandemic*” and “*The study conditions (online classes) in my subject area are frustrating*”). In our work, we used an overall measure of study satisfaction encompassing all three subscales. Relations to key criteria (e.g., study motivation, conscientiousness) are provided, suggesting convergent validity evidence (Westermann et al., 2018). Participants answered on a 101-point Likert scale ranging from 0% (*not satisfied at all*) to 100% (*completely satisfied*).

#### Coping

To assess coping with the COVID-19 pandemic, we used a German translation (Kälin, 1994) of the Brief COPE (Carver, 1997) and adapted it slightly. Specifically, we added a time frame and coping target to the item instructions: “*Since last lockdown in December 2020 to cope with the COVID-19 pandemic,* …” The 28-item Brief COPE consists of 14 subscales with two items per scale which we aggregated to four overarching dimensions in accordance with more recent works on its factorial structure (e.g., Folkman and Moskowitz, 2004; Nahlen Bose et al., 2015; Baumstarck et al., 2017).

The first dimension or scale, respectively, was active coping which included the subscales active coping (e.g., “*I’ve been taking action to try to make the situation better*”) and planning (e.g., “*I’ve thinking hard about what steps to take*”). The second scale was avoidance coping which included the subscales self-distraction (e.g., “*I’ve been turning to work or other activities to take my mind off things*”), denial (e.g., “*I’ve been saying to myself ‘this is not real’*”), substance use (e.g., “*I’ve been using alcohol or other drugs to make myself feel better*”), behavioral disengagement (e.g., “*I’ve been giving up trying to deal with it*”), and self-blame (e.g., “*I’ve been criticizing myself*”). The third scale was social support and included the subscales seeking emotional support (e.g., “*I’ve been getting emotional support from others*”), seeking instrumental support (e.g., “*I’ve been getting help and advice from other people*”), and venting (e.g., “*I’ve been expressing my negative feelings*”). Finally, the fourth scale was positive cognitive restructuring and included the subscales positive cognitive restructuring (e.g., “*I’ve been looking for something good in what’s happening*”), humor (e.g., “*I’ve been making jokes about it*”), and acceptance (e.g., “*I’ve been accepting the reality of the fact that it has happened*”). Participants responded on a five-point Likert scale ranging from 1 (*not at all*) to 5 (*a lot*), such that higher ratings indicated a more frequent use of the respective coping strategies. The subscale religion (e.g., “*I’ve been praying or meditating*”) was omitted due to little variance in our sample. Baumstarck et al. (2017) reported internal consistencies of the four scales ranging between *α* = .64 and *α* = .82 as well as external validity evidence in the form of relations to significant criteria (e.g., quality of life, mental health).

#### Outcomes

##### Loneliness

Loneliness was assessed with the German version (Huxhold et al., 2019) of the 6-item De Jong Gierveld Loneliness Scale (De Jong Gierveld and van Tilburg, 2006) that measures the two components social (i.e., missing a social network) and emotional (i.e., missing an intimate relationship) loneliness. Again, we adapted item instructions to refer to the same time frame during the COVID-19 pandemic: “*Please indicate to which extent you agree with the following statements since last lockdown in December 2020.*” Example items are “*I experience a general sense of emptiness*” (for emotional loneliness) and “*There are plenty of people I can rely on when I have problems*” (for social loneliness, negative indicator). In the German-language loneliness scale, a four-point Likert scale is used, ranging from 1 (*not at all*) to 4 (*exactly*), such that higher ratings indicate higher loneliness. Reliability estimates ranged from *α* = .67 to *α* = .74 for the emotional loneliness subscale, and from *α* = .69 to *α* = .73 for the social loneliness subscale (De Jong Gierveld and van Tilburg, 2006). Validity evidence in the form of correlations with loneliness determinants (i.e., partner status and subjective health) were provided (De Jong Gierveld and van Tilburg, 2006).

##### Life Satisfaction

Life satisfaction was measured as cognitive aspect of subjective well-being using a single-item scale (Beierlein et al., 2015). We adapted the item wording to refer to the same time frame during the COVID-19 pandemic; i.e., “*How satisfied are you since last lockdown in December 2020, all in all, with your life?*.” Participants responded on a 10-point Likert scale ranging from 1 (*not satisfied at all*) to 10 (*completely satisfied*) such that higher ratings indicate higher life satisfaction. Beierlein et al. (2015) reported an average test–retest reliability of *α* = .67 across 6 weeks, indicating sufficient reliability for group-level analyses. Moreover, a positive correlation between this single-item scale and the well-known five-item satisfaction with life scale by Diener et al. (1985) of *r* = .74 was reported, suggesting convergent validity (Beierlein et al., 2015).

##### COVID-19-Related Stress

To assess subjective COVID-19-related stress, we used two self-developed items. We conceptualized COVID-19 stress as an indicator of the perceived psychological burden caused by the COVID-19 pandemic in general as well as the burden by the government-implemented measures to mitigate the spread of the virus: “*How burdened do you feel with the COVID-19 pandemic?*” and “*How burdened do you feel with the measures undertaken to contain the COVID-19 pandemic and their related restrictions?*.” Again, participants were asked to refer to the time frame since last lockdown in December 2020. Participants responded on a 10-point Likert scale ranging from 1 to 10 with no verbal anchor points, such that higher ratings represented higher COVID-19-related stress.

### Statistical Analyses

For statistical analyses we used the statistical software R (R Core Team, 2021) and specifically the package *lavaan* for mediation models (Rosseel, 2012). Before addressing our research questions, we examined descriptive statistics and psychometric properties of all our measures across both measurement points t_1_ and t_2_. Due to non-skippable items in the questionnaire, we had 0% of missing data across all items. Since the metrics of our variables varied substantially, we scaled all variables to have a mean of zero and a standard deviation of 1 prior to conducting our analyses.

To estimate the cross-sectional effects of individual and organizational soldier student factors on loneliness, life satisfaction, and COVID-19 stress (RQ1), we computed multiple linear regressions for each outcome variable that were carried out in a stepwise manner. Specifically, we entered gender, age, and days spent in the barracks in the previous month as covariates in step 1, then added the three personality traits (i.e., extraversion, neuroticism, and conscientiousness) as predictors in step 2, and finally added the two organizational factors (i.e., commitment and study satisfaction) as predictors in step 3. We then evaluated individual predictive effects as well as the change in effects by adding new predictors.

To estimate longitudinal effects of individual and organizational soldier student factors on change in loneliness, life satisfaction, and COVID-19 stress over 4 weeks (RQ2), we first examined the change in outcome variables from t_1_ to t_2_ using *t*-tests for paired samples. For effect size estimations, we calculated Cohen’s *d* and followed the conventional criteria (|*d*| ≥ .2 for a small effect, |*d*| ≥ .5 for a medium-sized effect, and |*d*| ≥ .8 for a large effect; Cohen, 1988). We subsequently performed multiple regression analyses using the three-step procedure described above with the following adjustments: We used predictors at t_1_, and created the difference value in each outcome variable (i.e., t_2_ − t_1_) to predict the *change* in loneliness, life satisfaction, and COVID-19 stress over the course of 4 weeks. Again, gender, age, and presence at campus were entered as covariates in step 1, personality traits as individual predictors in step 2, and commitment and study satisfaction as organizational predictors in step 3.

Finally, to address the question of whether different coping strategies mediate the relationships between individual and organizational soldier student factors and loneliness, life satisfaction, and COVID-19 stress (RQ3), we conducted mediation analyses. For the individual factors, we focused on coping dimensions that have been shown to be related to the different personality traits and psychological health in prior work (i.e., active coping and social support as mediators for the association between extraversion and psychological health, avoidant coping and social support for the neuroticism-psychological health link, and active coping and avoidant coping for the conscientiousness-psychological health relation). For the organizational factors, we used all four coping dimensions exploratively for both commitment and study satisfaction due to a lack of prior research in this field. Before conducting mediation analyses, we examined respective relations between predictor and mediator variables, and between mediator and outcome variables. Out of the set of planned mediations, we then only conducted those that showed significant direct relations to and from their potential mediator variable. We then conducted separate mediation analyses for each predictor and each outcome variable. The respective coping strategy was entered as mediator variable (and in the case of multiple mediator variables, these were entered simultaneously and allowed to correlate). In all models, we added gender, age, and presence at campus as covariates. We estimated the size of the indirect effects by calculating the squared standardized indirect path coefficients as an indicator of explained variance (Lachowicz et al., 2018). For cut-off-criteria we used 2% for a small effect, 15% for a medium-sized effect, and 25% for a large effect (Cohen, 1988).^4^

## Results

### Descriptive Statistics

The descriptive statistics of the implemented measures at both measurement points t_1_ and t_2_ are displayed in Table 2 along with the theoretical scale minima and maxima and coefficients of internal consistency and test–retest-reliability. To estimate internal consistencies, we calculated α coefficients that ranged between *α* = .62 and *α* = .89 across all measures and measurement points (see also Table 2). At the descriptive level, means and standard deviations were noticeably similar across measurement points for the individual and organizational factors, yet, somewhat lower for the coping strategies and the outcome variables loneliness, life satisfaction, and COVID-19 stress. This was reflected by indicators of test–retest reliability accordingly, where correlation coefficients ranged between *r* = .85 and *r* = .91 across individual and organizational factors, and between *r* = .46 and *r* = .72 across coping and outcome variables (all *p*s < .001).

**Table 2:** Descriptive statistics and *α* reliability coefficients.

	Theoretical min, max	t_1_			t_2_			Test–retest reliability t_1_ − t_2_
		*M*	*SD*	*α*	*M*	*SD*	*α*	*r*
**Individual factors (personality)**								
Extraversion	1, 5	3.57	.65	.86	3.55	.65	.89	.89^***^
Neuroticism	1, 5	2.66	.67	.87	2.51	.63	.88	.85^***^
Conscientiousness	1, 5	3.57	.62	.85	3.54	.67	.88	.91^***^
**Organizational factors**								
Commitment	1, 5	3.95	.82	.89	3.91	.78	.87	.88^***^
Study satisfaction	0, 100	56.47	21.82	.83	57.28	2.65	.81	.87^***^
**Mediators (coping strategies)**								
Active coping	1, 5	2.83	.91	.69	2.33	.84	.74	.46^***^
Avoidant coping	1, 5	1.87	.64	.81	1.70	.58	.82	.63^***^
Social support	1, 5	2.06	.86	.82	1.78	.72	.83	.56^***^
Positive cognitive restructuring	1, 5	3.46	.74	.69	3.35	.72	.62	.61^***^
**Outcomes**								
Social loneliness	1, 4	1.65	.65	.81	1.56	.59	.80	.58^***^
Emotional loneliness	1, 4	2.37	.79	.70	2.24	.80	.79	.72^***^
Life satisfaction	1, 10	6.69	2.00	---	7.13	1.52	---	.64^***^
COVID-19 stress	1, 10	5.43	2.35	.83	4.25	2.10	.78	.69^***^

Min, minimum; Max, maximum; M, mean; and SD, standard deviation. *N* of sample t_1_ = 106 and *N* of Sample t_2_ = 63.

* *p* < .05;

** *p* < .01;

*** *p* < .001.

To gain some first insight into the specifics of our soldier student sample, we compared our mean levels (using t_1_ as reference) to the ones reported in other samples before the pandemic descriptively. If scale ranges differed across studies, they were transformed into a common scale of 0 to 100 to be comparable. While extraversion was slightly more pronounced in our sample (Δ*M* = .35), levels of neuroticism (Δ*M* = −.06) and conscientiousness (Δ*M* = −.10 on a scale of 1–5, respectively) were comparable to a representative German sample (Danner et al., 2016). Affective organizational commitment was higher than in a comparable sample of German soldier students from 2011 (Δ*M* = .20 on a scale of 1–5; Felfe and Scherm, 2012). Study satisfaction during the COVID-19 pandemic in our sample was lower (Δ*M* = −11.70 on a scale of 0–100) than study satisfaction of a German student sample prior to the pandemic (Bernholt et al., 2018). On a scale of 0–100, the use of active (Δ*M* = 10.90) and avoidant coping (Δ*M* = 7.10) were somewhat more frequent in our sample, while seeking support was comparable across samples (Δ*M* = −2.30), whereas positive cognitive restructuring was reported to be used more frequently compared to the other strategies within our sample and compared to other samples (Δ*M* = 23.30; Baumstarck et al., 2017). A loneliness value of >2.5 was reported to be indicative of substantial loneliness (Huxhold et al., 2019), which was not the case in our sample, although our sample showed increased emotional loneliness that approximated the cut-off value as compared to social loneliness. Life satisfaction in our sample was higher (Δ*M* = 9.26 on a scale of 0–100) compared to a German pre-pandemic sample in an online survey (Beierlein et al., 2015). COVID-19 stress values in our sample resembled responses obtained from another German sample with a similar scale during the pandemic (Δ*M* = −.84 on a scale of 1–100; Brailovskaia et al., 2021). Note that the relations between COVID-19 stress and other measures used in the present study that are described below (e.g., positive relations between COVID-19 stress and neuroticism) can be interpreted as validity evidence of the newly developed measure. Taken together, at the descriptive level, our soldier student sample showed more pronounced extraversion, commitment, active and avoidant coping, positive cognitive restructuring, and life satisfaction scores, while ratings of study satisfaction were lower as compared to other samples before the pandemic. With the exception of commitment, the comparison samples were not soldier students, such that any differences might result from the specific soldier student sample, or the pandemic circumstances (or both).

Construct correlations within measurement points (i.e., cross-sectional correlations) can be found in Table 3. Correlations among personality traits were as expected, displaying negative relations between neuroticism and both extraversion and conscientiousness (ranges from *r* = −.41 to *r* = −.28, all *p*s < .01), and positive relations between extraversion and conscientiousness (*r* = .31 and *r* = .34 at t_1_ and t_2_, respectively). Correlations among the different coping strategies showed positive relations between active coping, avoidant coping, and social support (ranges from *r* = .31 to *r* = .61, all *p*s < .01), while positive cognitive restructuring was unrelated to either of the three. Correlations among the loneliness measures were as expected, indicating a positive relation between social and emotional loneliness with *r* = .34 and *r* = .49 (*p*s < .001).

**Table 3:** Correlations within measurement points.

Variable	E	N	C	Com	StSa	AcCo	AvCo	SeSu	PoTh	SoLo	EmLo	LiSa	CoStr
**t**_1_													
E	---												
N	−.28^**^	---											
C	.31^**^	−.32^***^	---										
Com	.16	−.25^*^	−.07	---									
StSa	.30^**^	−.49^***^	.25^**^	.01	---								
AcCo	.26^**^	.06	.07	.05	−.01	---							
AvCo	−.13	.52^***^	−.37^***^	−.18	−.42^***^	.31^**^	---						
SeSu	.04	.48^***^	−.08	−.06	−.32^***^	.44^***^	.59^***^	---					
PoTh	.17	−.24^*^	.02	.12	.31^**^	.17	−.07	−.17	---				
SoLo	−.27^**^	.38^***^	.01	−.37^***^	−.24^*^	−.01	.30^*^	.18	−.17	---			
EmLo	−.09	.37^***^	−.13	.03	−.45^***^	.26^**^	.47^***^	.50^***^	−.22^*^	.34^***^	---		
LiSa	.25^*^	−.52^***^	.19	.26^**^	.55^***^	−.06	−.53^***^	−.30^**^	.20^*^	−.46^***^	−.47^***^	---	
CoStr	.06	.42^***^	−.13	−.10	−.26^**^	.40^***^	.58^***^	.52^***^	−.17	.27^**^	.58^***^	−.39^***^	---
**t**_2_													
E	---												
N	−.33^**^	---											
C	.34^**^	−.41^***^	---										
Com	.19	−.15	−.14	---									
StSa	.26^*^	−.41^***^	.43^***^	−.05	---								
AcCo	.17	.25^*^	−.28^*^	.03	−.16	---							
AvCo	−.01	.44^***^	−.53^***^	.12	−.43^***^	.61^***^	---						
SeSu	.02	.42^***^	−.32^*^	.16	−.39^**^	.59^***^	.60^***^	---					
PoTh	.10	−.22	.24	−.02	.16	.04	−.03	−.13	---				
SoLo	−.23	.46^***^	−.19	−.07	−.21	.20	.23	.22	−.19	---			
EmLo	.03	.35^**^	−.23	.19	−.46^***^	.32^*^	.38^*^	.52^***^	−.23	.49^***^	---		
LiSa	.33^**^	−.38^**^	.25	.02	.41^***^	.04	−.27^*^	−.24	.28^*^	−.10	−.22	---	
CoStr	.23	.29^*^	−.23	.12	−.24	.41^***^	.41^***^	.52^***^	−.32^**^	.18	.56^***^	−.13	---

E, extraversion; N, neuroticism; C, conscientiousness; Com, commitment; StSa, study satisfaction; AcCo, active coping; AvCo, avoidant coping; SeSu, social support; PoTh, positive cognitive restructuring; SoLo, social loneliness; EmLo, emotional loneliness; LiSa, life satisfaction; and CoStr, COVID-19 stress. *N* of Sample t_1_ = 106 and N of Sample t_2_ = 63.

* *p* < .05;

** *p* < .01;

*** *p* < .001.

Relations between individual and organizational predictor variables, potentially mediating coping strategies, and outcome variables generally reflected theoretical and empirical expectations. Extraversion was positively related to study satisfaction, active coping at t_1_, and life satisfaction (ranges from *r* = .26 to *r* = .33, *p*s < .05), and negatively related to social loneliness at t_1_ (*r* = −.27, *p* < .01). Neuroticism showed negative relations to commitment at t_1_, study satisfaction, and life satisfaction (ranges from *r* = −.49 to *r* = −.25, *p*s < .05) and positive relations to avoidant coping, social support, social and emotional loneliness and COVID-19 stress (ranges from *r* = .25 to *r* = .52, *p*s < .05), but also to active coping at t_2_ (*r* = .25, *p* < .05). Conscientiousness was positively related to study satisfaction (*r* = .25 and *r* = .43, *p*s < .01), and negatively related to avoidant coping and social support at t_2_ (ranges from *r* = −.32 to *r* = −.53, *p*s < .05), but also to active coping at t_2_ (*r* = −.28, *p* < .05). Commitment showed a negative relation to social loneliness at t_1_ (*r* = −.37, *p* < .001) and positive relations to life satisfaction at t_1_ (*r* = .26, *p* < 01). Study satisfaction was negatively related to avoidant coping, social support, social (t_1_) and emotional loneliness, and COVID-19 stress at t_1_ (ranges from *r* = −.45 to *r* = −.26, *p*s < .01), while showing positive relations to positive cognitive restructuring at t_1_ and life satisfaction (ranges from *r* = .31 to *r* = .55, *p*s < .01). Active coping showed unexpected positive relations to emotional loneliness and COVID-19 stress (ranges from *r* = .26 to *r* = .41, *p*s < .05). Avoidant coping showed expected positive relations to social (t_1_) and emotional loneliness and COVID-19 stress (ranges from *r* = .30 to *r* = .58, *p*s < .05), and negative relations to life satisfaction (*r* = −.53 and *r* = −.27, *p*s < .05). Social support displayed a similar pattern with positive relations to emotional loneliness and COVID-19 stress (ranges from *r* = .50 to *r* = .52, *p*s < .001), and negative relations to life satisfaction at t_1_ (*r* = −.30, *p* < .01). Finally, positive cognitive restructuring showed negative relations to emotional loneliness at t_1_ and COVID-19 stress at t_2_ (*r* = −.22 and *r* = −.32, *p*s < .05), and positive relations to life satisfaction (*r* = .20 and *r* = .28, *p*s < .05).

Correlations across measurement points (i.e., longitudinal correlations) are shown in Table A1.

### Direct Cross-Sectional Effects of Individual and Organizational Factors (RQ1)

We first addressed direct cross-sectional effects of individual and organizational soldier student factors on loneliness, life satisfaction, and COVID-19 stress (RQ1). Results of the multiple regressions that were carried out in three steps can be found in Table 4. The covariates gender, age, and presence at campus showed effects in only two cases across all models (i.e., age predicted emotional loneliness negatively at t_2_, *β* = −.28, *p* < .05, and gender predicted COVID-19 stress negatively at t_1_, *β* = −.23, *p* < .05, indicating higher COVID-19 stress for females. Neither of these effects remained statistically significant when personality traits were entered as predictors in step 2 suggesting a better predictability of relations through personality traits than age and gender).

**Table 4:** Standardized path coefficients of direct cross-sectional multiple regression models.

	t_1_			t_2_		
	Step 1: *β* (*SE*)	Step 2: *β* (*SE*)	Step 3: *β* (*SE*)	Step 1: *β* (*SE*)	Step 2: *β* (*SE*)	Step 3: *β* (*SE*)
Predictor at t_1_ or t_2_	Outcome: social loneliness at t_1_			Outcome: social loneliness at t_2_		
Gender	−.13 (0.10)	.00 (0.10)	.01 (0.10)	.01 (0.13)	.00 (0.13)	.00 (0.14)
Age	.01 (0.10)	−.02 (0.09)	−.06 (0.09)	−.20 (0.15)	−.10 (0.14)	−.10 (0.16)
Presence at campus	.06 (0.10)	.05 (0.09)	.06 (0.09)	−.12 (0.13)	−.08 (0.12)	−.08 (0.13)
Extraversion	---	−.23^*^ (0.10)	−.17 (0.10)	---	−.08 (0.13)	−.08 (0.13)
Neuroticism	---	.37^***^ (0.10)	.27^*^ (0.11)	---	.41^**^ (0.14)	.39^**^ (0.15)
Conscientiousness	---	.20 (0.10)	.16 (0.10)	---	.00 (0.14)	.00 (0.15)
Commitment	---	---	−.28^**^ (0.09)	---	---	−.02 (0.14)
Study satisfaction	---	---	−.08 (0.11)	---	---	−.03 (0.14)
	Outcome: emotional loneliness at t_1_			Outcome: emotional loneliness at t_2_		
Gender	−.13 (0.10)	−.05 (0.10)	−.09 (0.10)	−.03 (0.13)	−.08 (0.13)	−.10 (0.13)
Age	−.07 (0.10)	−.08 (0.09)	−.07 (0.09)	−.28^*^ (0.14)	−.20 (0.14)	−.13 (0.14)
Presence at campus	.19 (0.10)	.17 (0.09)	.10 (0.09)	.03 (0.13)	.05 (0.13)	−.01 (0.12)
Extraversion	---	.03 (0.10)	.07 (0.10)	---	.19 (0.13)	.20 (0.12)
Neuroticism	---	.36^***^ (0.10)	.22 (0.11)	---	.30^*^ (0.14)	.22 (0.14)
Conscientiousness	---	.00 (0.11)	.01 (0.10)	---	−.17 (0.14)	−.04 (0.14)
Commitment	---	---	.06 (0.09)	---	---	.12 (0.13)
Study Satisfaction	---	---	−.35^**^ (0.10)	---	---	−.40^**^ (0.13)
	Outcome: life satisfaction at t_1_			Outcome: life satisfaction at t_2_		
Gender	.12 (0.10)	.01 (0.09)	.04 (0.08)	.16 (0.14)	.23 (0.13)	.25 (0.13)
Age	.05 (0.10)	.07 (0.09)	.09 (0.08)	.01 (0.15)	−.09 (0.14)	−.14 (0.15)
Presence at campus	−.15 (0.10)	−.12 (0.09)	−.06 (0.08)	−.10 (0.14)	−.13 (0.12)	−.08 (0.12)
Extraversion	---	.11 (0.09)	.02 (0.08)	---	.21 (0.13)	.21 (0.13)
Neuroticism	---	−.50^***^ (0.09)	−.26^**^ (0.10)	---	−.27 (0.14)	−.21 (0.14)
Conscientiousness	---	−.02 (0.10)	.00 (0.09)	---	.13 (0.14)	.04 (0.15)
Commitment	---	---	.20^*^ (0.08)	---	---	−.08 (0.13)
Study satisfaction	---	---	.40^***^ (0.09)	---	---	.28^*^ (0.13)
	Outcome: COVID-19 stress at t_1_			Outcome: COVID-19 stress at t_2_		
Gender	−.23^*^ (0.10)	−.16 (0.10)	−.17 (0.10)	.09 (0.13)	.06 (0.13)	.05 (0.13)
Age	−.08 (0.10)	−.08 (0.09)	−.08 (0.09)	−.23 (0.15)	−.17 (0.14)	−.16 (0.15)
Presence at campus	.08 (0.10)	.07 (0.09)	.04 (0.09)	−.01 (0.13)	−.01 (0.12)	−.03 (0.12)
Extraversion	---	.20^*^ (0.09)	.23^*^ (0.10)	---	.42^**^ (0.12)	.43^**^ (0.13)
Neuroticism	---	.41^***^ (0.10)	.34^**^ (0.11)	---	.30^*^ (0.13)	.26 (0.14)
Conscientiousness	---	−.09 (0.10)	−.09 (0.10)	---	−.22 (0.14)	−.18 (0.15)
Commitment	---	---	−.05 (0.10)	---	---	−.01 (0.13)
Study satisfaction	---	---	−.13 (0.11)	---	---	−.14 (0.14)

SE, standard error. *N* of sample t_1_ = 106 and *N* of sample t_2_ = 63.

* *p* < .05;

** *p* < .01;

*** *p* < .001.

Within each of the two measurement points, a, respectively, similar result pattern emerged. Neuroticism showed to be the strongest predictor among the personality traits, displaying positive relations to emotional and social loneliness, and COVID-19 stress (ranges from *β* = .30 to *β* = .42, *p*s < .05), and a negative relation to life satisfaction (*β* = −.50, *p* < .001) in step 2. Extraversion predicted social loneliness negatively (*β* = −.23, *p* < .05) and COVID-19 stress positively (*β* = .20 and *β* = .42, *p* < .05) in step 2. However, after the inclusion of commitment and study satisfaction in step 3, most of these effects either shrunk in size, or did not remain statistically significant. Instead, commitment predicted social loneliness at t_1_ negatively (*β* = −.28, *p* < .01) and life satisfaction at t_1_ positively (*β* = .20, *p* < .05) over and above the personality traits. Study satisfaction predicted emotional loneliness negatively (*β* = −.35 and *β* = −.40, *p* < .01), and life satisfaction positively (*β* = .40 and *β* = .28, *p* < .05). In conclusion, more extraverted people tended to experience more COVID-19 stress. Persons who scored higher on neuroticism tended to show associations with more negatively connotated (i.e., loneliness and COVID-19 stress) and less positively connotated variables (i.e., life satisfaction). The expected relations presented in Table 1 were partially supported. Contrary to expectations, extraversion and conscientiousness were unrelated to loneliness and life satisfaction, and conscientiousness was unrelated to COVID-19 stress as well. Commitment was negatively related to social but not emotional loneliness, and vice versa for study satisfaction, while both were unrelated to COVID-19 stress. All other relations were in the expected direction. Although neuroticism was the strongest predictor among all three personality traits, the organizational factors commitment and study satisfaction showed incremental validity in explaining some of these outcome variables above and beyond personality traits (RQ1).

### Direct Longitudinal Effects of Individual and Organizational Factors (RQ2)

We then addressed the question of direct longitudinal effects of individual and organizational factors on the change in loneliness, life satisfaction, and COVID-19 stress (RQ2). Means, standard deviations and results of paired samples *t*-tests along with their effect size are displayed in Table 5. The only statistically significant changes from t_1_ to t_2_ were found for emotional loneliness, *t*(62) = 2.11, *p* < .05, *d* = .27, and for COVID-19 stress *t*(62) = 5.39, *p* < .001, *d* = .68, which both decreased over time. The stepwise multiple regression analyses with the difference value in each outcome variable (i.e., the change values) did not reveal any significant relations for individual and organizational factors across all models (see Table 5). Gender showed to be a significantly positive predictor of change in COVID-19 stress that remained significant after the inclusion of all individual and organizational predictors (*β* = .34, *p* < .05), indicating that females reported a greater decrease in COVID-19 stress than males. An additional *t*-test revealed that at t_1_, females had significantly higher COVID-19 stress (*M* = 6.00, *SD* = 2.16) than males (*M* = 4.92, *SD* = 2.40), *t*(104) = 2.44, *p* < .05, while the COVID-19 stress at t_2_ did not differ across gender anymore, *t*(58) = −.32, *p* > .05. Taken together, results indicated that participants experienced substantial changes in emotional loneliness and COVID-19 stress between the measurement points. Yet, these changes could not be predicted by individual or organizational soldier student factors, while the change in COVID-19 stress could be predicted by gender.

**Table 5:** Descriptive statistics, *t*-tests of change, and standardized path coefficients of direct longitudinal multiple regression models.

	***M***_**t2-t1**_	***SD***_**t2-t1**_	***t* (df)**	***d***
Change in social loneliness from t_1_ to t_2_	−.02	.53	.32 (62)	.04
Change in emotional loneliness from t_1_ to t_2_	−.15	.58	2.11^*^ (62)	.27
Change in life satisfaction from t_1_ to t_2_	.22	1.35	−1.31 (62)	−.17
Change in COVID-19 stress from t_1_ to t_2_	−1.18	1.74	5.39^***^ (62)	.68
	**Step 1: *β* (SE)**	**Step 2: *β* (SE)**	**Step 3: *β* (SE)**	
Predictor at t_1_	Outcome: change in social loneliness			
Gender	.04 (0.14)	.03 (0.15)	.03 (0.15)	
Age	−.15 (0.15)	−.13 (0.15)	−.06 (0.16)	
Presence at campus	−.14 (0.14)	−.12 (0.14)	−.15 (0.14)	
Extraversion	---	.17 (0.14)	.12 (0.14)	
Neuroticism	---	.16 (0.15)	.20 (0.17)	
Conscientiousness	---	−.05 (0.15)	.01 (0.16)	
Commitment	---	---	.24 (0.16)	
Study satisfaction	---	---	.04 (0.16)	
	Outcome: change in emotional loneliness			
Gender	−.04 (0.14)	−.04 (0.15)	−.05 (0.16)	
Age	−.06 (0.15)	−.06 (0.15)	−.04 (0.16)	
Presence at campus	.08 (0.14)	.09 (0.14)	.08 (0.14)	
Extraversion	---	.19 (0.14)	.19 (0.15)	
Neuroticism	---	.18 (0.15)	.17 (0.17)	
Conscientiousness	---	−.04 (0.15)	−.02 (0.16)	
Commitment	---	---	.04 (0.17)	
Study satisfaction	---	---	−.05 (0.17)	
	Outcome: change in life satisfaction			
Gender	.20 (0.13)	.20 (0.15)	.16 (0.15)	
Age	−.15 (0.15)	−.13 (0.15)	−.16 (0.16)	
Presence at campus	−.06 (0.13)	−.04 (0.14)	−.05 (0.14)	
Extraversion	---	.00 (0.14)	.08 (0.14)	
Neuroticism	---	.16 (0.15)	.05 (0.16)	
Conscientiousness	---	−.03 (0.15)	−.05 (0.16)	
Commitment	---	---	−.19 (0.16)	
Study satisfaction	---	---	−.20 (0.16)	
	Change in COVID-19 stress			
Gender	.29^*^ (0.13)	.34^*^ (0.15)	.34^*^ (0.15)	
Age	−.05 (0.15)	−.07 (0.15)	−.13 (0.16)	
Presence at campus	−.01 (0.13)	−.01 (0.13)	.02 (0.14)	
Extraversion	---	.03 (0.13)	.07 (0.14)	
Neuroticism	---	.04 (0.15)	.02 (0.17)	
Conscientiousness	---	.11 (0.15)	.06 (0.16)	
Commitment	---	---	−.19 (0.16)	
Study satisfaction	---	---	.05 (0.16)	

SD, standard deviation; *d*, Cohen’s *d*; and SE, standard error. *N* of sample t_1_ = 106; *N* of sample t_2_ = 63.

* *p* < .05;

** *p* < .01;

*** *p* < .001.

### Mediation Effects Through Coping (RQ3)

Finally, we addressed the question of coping strategies as possible mediators of the relationship between individual and organizational soldier student factors and loneliness, life satisfaction, and COVID-19 stress (RQ3). For the individual factors, we aimed at testing specific relations (see section Statistical Analyses), while we aimed at testing all four coping dimensions exploratively for the organizational factors. As a prerequisite for mediation analyses, we first examined relations of individual and organizational factors to their respective mediators as well as relations of the potential mediators to the outcome variables. Not all postulated direct relations reached statistical significance at either of the measurement points (see Table 3). Specifically, relations between extraversion and social support, between commitment and all four coping strategies, between active coping and study satisfaction, social loneliness, and life satisfaction, between social support and social loneliness, and between positive cognitive restructuring and social loneliness were not statistically different from zero (see Table 3) which was partially in contrast to previous expectations (Table 1). Therefore, mediation analyses building upon either of these relations were not conducted.

Standardized indirect effects for mediation effects between individual (i.e., personality) factors and outcome variables are shown in Table 6. In general, more statistically significant paths can be seen at t_1_ as opposed to t_2_. Avoidant coping was the most frequent mediator at t_1_, mediating between neuroticism and life satisfaction (*β* = −.21, *p* < .01), neuroticism and COVID-19 stress (*β* = .23, *p* < .001), conscientiousness and social loneliness (*β* = −.13, *p* < .05), conscientiousness and life satisfaction (*β* = .20, *p* < .01), and conscientiousness and COVID-19 stress (*β* = −.20, *p* < .05). Active coping mediated the relation between extraversion and COVID-19 stress only (*β* = .10, *p* < .05). Social support mediated the relation between neuroticism and emotional loneliness (*β* = .15, *p* < .01) and neuroticism and COVID-19 stress (*β* = .10, *p* < .05). At t_2_, only social support significantly mediated relations between neuroticism and emotional loneliness, and neuroticism and COVID-19 stress, respectively (*β* = .16 and *β* = .18, *p* < .05).

**Table 6:** Standardized indirect path coefficients, standard errors, and effect sizes for the individual factor-mediation models.

	t_1_		t_2_	
	*β*_ind_ (*SE*)	*β*_ind_^2^	*β*_ind_ (*SE*)	*β*_ind_^2^
**Predictor: extraversion**	Outcome: emotional loneliness			
Extraversion ➔ Active coping ➔ Emotional loneliness	.07 (0.04)	.00	.05 (0.04)	.00
	Outcome: COVID-19 stress			
Extraversion ➔ Active coping ➔ COVID-19 stress	.10^*^ (0.04)	.01	.06 (0.05)	.00
**Predictor: neuroticism**	Outcome: social loneliness			
Neuroticism ➔ Avoidant coping ➔ Social loneliness	.08 (0.06)	.01	.01 (0.05)	.00
	Outcome: emotional loneliness			
Neuroticism ➔ Avoidant coping ➔ Emotional loneliness	.11 (0.06)	.01	.04 (0.06)	.00
Neuroticism ➔ Social support ➔ Emotional loneliness	.15^**^ (0.06)	.03	.16^*^ (0.07)	.03
	Outcome: life satisfaction			
Neuroticism ➔ Avoidant coping ➔ Life satisfaction	−.21^**^ (0.06)	.05	−.10 (0.07)	.01
Neuroticism ➔ Social support ➔ Life satisfaction	.07 (0.05)	.00	.00 (0.06)	.00
	Outcome: COVID-19 stress			
Neuroticism ➔ Avoidant coping ➔ COVID-19 stress	.23^***^ (0.06)	.05	.05 (0.06)	.00
Neuroticism ➔ Social support ➔ COVID-19 stress	.10^*^ (0.05)	.01	.18^*^ (0.08)	.03
**Predictor: conscientiousness**	Outcome: social loneliness			
Conscientiousness ➔ Avoidant coping ➔ Social loneliness	−.13^**^ (0.05)	.02	−.08 (0.08)	.01
	Outcome: emotional loneliness			
Conscientiousness ➔ Active coping ➔ Emotional loneliness	.01 (0.01)	.00	−.04 (0.04)	.00
Conscientiousness ➔ Avoidant coping ➔ Emotional loneliness	−.16^**^ (0.05)	.03	−.13 (0.09)	.02
	Outcome: life satisfaction			
Conscientiousness ➔ Avoidant coping ➔ Life satisfaction	.20^**^ (0.06)	.04	.14 (0.08)	.02
	Outcome: COVID-19 stress			
Conscientiousness ➔ Active coping ➔ COVID-19 stress	.01 (0.02)	.00	−.08 (0.05)	.01
Conscientiousness ➔ Avoidant coping ➔ COVID-19 stress	−.20^**^ (0.06)	.04	−.11 (0.09)	.01

SE, standard error. *N* of sample t_1_ = 106 and *N* of sample t_2_ = 63.

* *p* < .05;

** *p* < .01;

*** *p* < .001.

Standardized indirect effects for organizational factors and outcome variables are shown in Table 7. Mediation models with commitment as predictor were omitted due to the lack of significant relations of commitment to either of the coping strategies. For study satisfaction as predictor, the pattern of results differed between the two measurement points. Again, avoidant coping dominantly mediated relations at t_1_, specifically the relation between study satisfaction and social loneliness (*β* = −.10, *p* < .05), study satisfaction and life satisfaction (*β* = .18, *p* < .01), and study satisfaction and COVID-19 stress (*β* = −.20, *p* < .01), while social support mediated the relation between study satisfaction and emotional loneliness (*β* = −.09, *p* < .05). At t_2_, the pattern changed such that social support was the only coping strategy showing significant indirect effects, mediating the relation between study satisfaction and emotional loneliness (*β* = −.13, *p* < .05) and study satisfaction and COVID-19 stress (*β* = −.16, *p* < .05). To sum up, we found significant indirect paths indicating mediation effects, i.e., indications of mechanisms underlying the personality-psychological health and study satisfaction-psychological health associations through certain coping strategies. The result pattern largely differed between the two measurement points. Avoidant coping and social support mainly mediated relations between personality traits and study satisfaction on the one hand and loneliness, life satisfaction, and COVID-19 stress on the other hand, with avoidant coping focally mediating relations at t_1_, and social support focally mediating relations at t_2_.

**Table 7:** Standardized indirect path coefficients, standard errors, and effect sizes for the organizational factor-mediation models.

	t_1_		t_2_	
	*β*_ind_ (*SE*)	*β*_ind_^2^	*β*_ind_ (*SE*)	*β*_ind_^2^
**Predictor: study satisfaction**	Outcome: social loneliness			
Study satisfaction ➔ Avoidant coping ➔ Social loneliness	−.10^*^ (0.05)	.01	−.07 (0.06)	.00
	Outcome: emotional loneliness			
Study satisfaction ➔ Avoidant coping ➔ Emotional loneliness	−.07 (0.05)	.00	−.03 (0.06)	.00
Study satisfaction ➔ Social support ➔ Emotional loneliness	−.09^*^ (0.04)	.01	−.13^*^ (0.06)	.02
Study satisfaction ➔ Positive cognitive restructuring ➔ Emotional loneliness	−.02 (0.03)	.00	−.02 (0.02)	.00
	Outcome: life satisfaction			
Study satisfaction ➔ Avoidant coping ➔ Life satisfaction	.18^**^ (0.06)	.03	.10 (0.07)	.01
Study satisfaction ➔ Social support ➔ Life satisfaction	−.04 (0.03)	.00	−.02 (0.05)	.00
Study satisfaction ➔ Positive cognitive restructuring ➔ Life satisfaction	.02 (0.03)	.00	.04 (0.03)	.00
	Outcome: COVID-19 stress			
Study satisfaction ➔ Avoidant coping ➔ COVID-19 Stress	−.20^**^ (0.06)	.04	−.06 (0.06)	.00
Study satisfaction ➔ Social support ➔ COVID-19 Stress	−.07 (0.04)	.00	−.16^*^ (0.07)	.03
Study satisfaction ➔ Positive cognitive restructuring ➔ COVID-19 stress	−.03 (0.03)	.00	−.05 (0.04)	.00

SE, standard error. *N* of sample t_1_ = 106 and *N* of sample t_2_ = 63. Significant indirect paths are printed in bold.

* *p < .05*;

** *p < .01*;

*** *p < .001.*

## Discussion

The COVID-19 pandemic has profoundly disrupted daily life and posed a significant multidimensional stressor for many people across the globe. Both students on the one hand and military personnel on the other hand have been reported to react to this extraordinary situation with psychological distress (e.g., Wang et al., 2020; Gordon et al., 2021). However, it is unclear how persons respond to the COVID-19 pandemic that comprise both roles—that of the student and of the soldier—at the same time. Therefore, the present study examined soldier students of the German *Bundeswehr* at two measurement points during the 2nd year of the COVID-19 pandemic in Germany. To consider this special organization, we investigated not only personality trait effects (extraversion, neuroticism, conscientiousness), but also organizational factor effects (organizational commitment and study satisfaction) on psychological health (loneliness, life satisfaction, and COVID-19 stress). We further considered potentially mediating effects of different coping dimensions (active coping, avoidant coping, seeking support, positive cognitive restructuring). In doing so, we aimed to highlight protective and vulnerability factors with regard to psychological health during the COVID-19 pandemic in a military student sample.

### Personality, Commitment, and Study Satisfaction as Predictors of Psychological Health

Personality traits, commitment, and study satisfaction showed high test–retest reliability coefficients, that exceeded those of social and emotional loneliness, life satisfaction, and COVID-19 stress at the descriptive level. Keeping in mind that all individual and organizational predictors (except for study satisfaction) were assessed in general (i.e., as traits), but all outcome variables were assessed with regard to a specific time frame during the pandemic, these findings might indicate the context-specificity in a dynamic situation. Other studies have already pointed out the need to closely monitor the respective time frame in such dynamic contexts with rapid changes in political measures (Moradian et al., 2021).

Results from the stepwise multiple regressions yielded support for the relevance of both personality traits and organizational factors in predicting psychological health indicators at the same time point (RQ1). Controlling for gender, age, and presence at campus, we observed positive relations between extraversion and COVID-19 stress that replicates previous reports of a weakened positive effect of extraversion on psychological health in times of social distancing (Gubler et al., 2021; Zacher and Rudolph, 2021a). Possibly, this might also be related to military samples that have shown to score higher in extraversion (Klee and Renner, 2016). Extraversion was unrelated to social and emotional loneliness and life satisfaction, when all other predictors were considered, suggesting a higher relevance of other variables. Neuroticism could be confirmed as a vulnerability factor for psychological stress, showing positive relations to social loneliness and negative relations to life satisfaction when all predictors were considered, thus replicating prior research. Conscientiousness was unrelated to either of the criteria. During the pandemic, conscientiousness mainly turned out to predict adherence to implemented containment-measures (Carvalho et al., 2020). The combination of higher conscientiousness scores in military samples (Klee and Renner, 2016) and the finding that adhering to measures has been shown to be related to higher depressive symptoms (Wright et al., 2021) might be the reason why conscientiousness seemed to have lost some of its positive impact on psychological health. Remarkably, the organizational factors commitment and study satisfaction were strong predictors over and above extraversion and neuroticism in social and emotional loneliness and life satisfaction, but not in COVID-19 stress. Commitment showed a negative relation to social loneliness and positive relation to life satisfaction above personality traits, standing in line with prior negative relations between commitment and stress in a military sample (Dobreva-Martinova, 2002). Study satisfaction was the only significant negative predictor of emotional loneliness, when all predictors were considered. Similar relations have been reported for job satisfaction (that we approximated with study satisfaction in our sample) and loneliness at the workplace (Tabancali, 2016; Bakır and Aslan, 2017). Moreover, study satisfaction predicted life satisfaction positively above all other predictors. Such a spillover effect has been shown in the literature already (Heller et al., 2004). Yet, this relation could also be explained by a higher perceived job importance in the military sector during the pandemic that has been identified as moderator of job and life satisfaction in prior work (Rice et al., 1985). Taken together, we identified relations of personality traits to psychological health outcomes, but also relations of organizational factors that showed incremental validity in predicting psychological health outcomes. This indicates a high relevance of organizational factors for psychological health of soldier students who live and study in barracks at campus.

Examining relations between individual and organizational predictors and the change in psychological health outcomes over 4 weeks (RQ2) did not yield any significant results except for a gender effect on the change in COVID-19 stress (i.e., females experiencing a stronger decrease in COVID-19 stress over time than males). Females had reported significantly higher COVID-19 stress than males at the first but not at the second measurement point. Previous research reported higher stress levels during the pandemic for females (Prowse et al., 2021; Peyer et al., 2022). Our findings thus indicate the advantage of longitudinal measurement that allows for estimating the stability or variability of differences.

Individual and organizational factors were not able to predict change in any of the psychological health outcomes. Considering the large autocorrelations of the outcome variables between the two measurement points, it seems plausible to assume that the variance of change is limited (i.e., the variables were too stable), motivating research on possibilities of initiating change in non-desirable outcomes (e.g., through tailored interventions). Future research could examine intraindividual change on a more fine-grained level (e.g., through daily diary studies) to reveal temporal dynamics and their antecedents and consequences.

### The Role of Coping Strategies in Predicting Psychological Health

When considering the role of coping strategies as possible mediators between individual and organizational predictors and psychological health outcomes (RQ3), we first registered some unexpected bivariate relations, including positive relations between active coping and avoidant coping, neuroticism, emotional loneliness, and COVID-19 stress, and negative relations of active coping to conscientiousness. Further, we found zero relations of positive cognitive restructuring to all other coping strategies. The accumulation of unexpected relations centering around the strategy active coping stands in contrast to previous findings that reported beneficial effects of active coping on psychological health during the pandemic (Budimir et al., 2021; Jin et al., 2021; Zacher and Rudolph, 2021b), yet referred to a different time frame. It is possible that in the 2nd year of the pandemic where our study was carried out, the phenomenon pandemic fatigue (Lilleholt et al., 2020) can account for these findings. Possibly, active coping strategies (e.g., planning) are inefficient when circumstances are highly unpredictable and change rapidly. Further, we found the use of social support to be positively related to emotional loneliness and COVID-19 stress, and negatively related to life satisfaction. This supports the notion of social support as “double-edged sword” (Carver et al., 1989; Revenson et al., 1991), that can highly differ in usefulness depending on the (dys-)functionality of social interactions. Contrary to previous findings, extraversion was not related to social support in our study. Further, commitment was unrelated to either of the coping strategies. Although the positive effect of commitment on psychological health is well established, the underlying mechanisms are hardly examined (Rivkin et al., 2018). In our study, we found no evidence for coping strategies as mechanisms of commitment’s effect on psychological health.

Evidence for mediation effects could be found for avoidant coping as mediator between neuroticism and both life satisfaction and COVID-19 stress, between conscientiousness and social loneliness, life satisfaction, and COVID-19 stress, between study satisfaction and social loneliness, life satisfaction, and COVID-19 stress. Active coping mediated the relation between extraversion and COVID-19 stress. Social support mediated relations between neuroticism and emotional loneliness and COVID-19 stress, and between study satisfaction and emotional loneliness and COVID-19 stress. We further found differences between the measurement points, such that avoidant coping dominantly mediated relations at the first measurement point while social support dominantly mediated relations at the second measurement point. We can thus conclude that coping strategies play an important role in processing stressful events and later psychological health in a military student sample. Among the coping strategies, avoidant coping and social support were the most pronounced mediators, while we, unexpectedly, did not find any indirect effects through positive cognitive restructuring. Differences between measurement points support the notion of coping as a *process* (Folkman and Moskowitz, 2004). The role of positive cognitive restructuring in our sample remains unclear, yet, it is descriptively reported to be the most frequently used coping strategy of all, and is further positively related to life and study satisfaction and negatively associated with COVID-19 stress. Perhaps, more stress-specific personality characteristics like sense of coherence (Antonovsky, 1993) or hardiness (Kobasa, 1979; Maddi, 2013) would operate as stronger predictors of this influential coping strategy (e.g., Williams et al., 1992; Pallant and Lae, 2002; Fok et al., 2005; Bartone and Bowles, 2020).

## Limitations

We note some important limitations of our study. First, within the cross-sectional analyses, we drew on a correlational design and used the terms of prediction and effect in accordance with theoretical assumptions, but of course not causality. Yet, we conducted a study with two measurement points over 4 weeks, that allows for (a) certain estimations of robustness of results, and (b) estimations of longitudinal effects controlling for previous construct manifestations. Second, our sample size particularly at the second measurement point, is limited, with about 60% of the initial sample partaking in the second measurement point. Consequentially, some regression coefficients at t_2_ are similar to coefficients at t_1_ in size, yet, do not reach statistical significance, partially accounting for differences in result patterns across the measurement points. Third, we conducted our study in an online format where inherently, a lack of control and insight on the response process and possible biases occurs. However, this was the most feasible way of data collection during the pandemic. Further, the online survey was thoroughly pre-tested to enhance user experience and prevent any systematic response bias. We found no indication for low data quality (e.g., extremely short response times) which was in line with prior research on the adequacy of online studies as compared to traditional methods (Gosling et al., 2004). Fourth, there is considerable incoherence in classifying individual coping strategies into higher-order coping strategies (Folkman and Moskowitz, 2004, see also Solberg et al., 2021 for a recent review). Further coping research could benefit significantly from coherent classifications that are derived from factorial validation efforts and are then comparable across studies. Fifth, we operationalized commitment and study satisfaction as organizational variables, although there is a confounding of individual and organizational aspects within these variables. We chose this approach as the *Bundeswehr* as military organization has well-known organizational features (Richter, 2017), and we considered individual perceptions of organizational well-being more crucial on psychological health outcomes. Nevertheless, further research could assess both self- and other-rated indicators of organizational features to obtain a more balanced picture.

## Implications and Conclusion

We found personality traits, but also organizational factors to be significantly related to psychological health (i.e., loneliness, life satisfaction, and COVID-19 stress), out of which some were mediated by different coping strategies in a sample of soldier students during the 2nd year of the COVID-19 pandemic. In doing so, we shed some light on stress and coping in a specific sample that comprises a professional double-role (i.e., soldier and student) in the German *Bundeswehr* during an extraordinary pandemic situation. These emergency service professions are pivotal in countries’ functioning in extreme cases. It is thus crucial to understand critical predictors of coping processes and psychological health in these groups. In the present study, we identified potentially vulnerability (i.e., neuroticism, extraversion, avoidant coping, active coping, social support) and protective factors (i.e., commitment, study satisfaction) in a pandemic setting and a military student sample. The relative importance of organizational variables *over and above* individual variables in this sample is remarkable. The findings of the present study yield important implications for the military in personnel selection and training. For instance, neuroticism showed to be a strong predictor of negative psychological health outcomes, enabling tailored and more efficient interventions even at the personality trait level (e.g., Roberts et al., 2017). Likewise, soldier students can be made aware of different coping strategies and the (mal-)adaptiveness under different circumstances such that they can engage in functional coping strategies that reduce psychological distress efficiently. These insights can be implemented in programs that lay emphasis on building strengths and personal resources to enhance employee well-being and psychological health (e.g., Krick et al., 2018). The predictive strength of affective organizational commitment and study satisfaction on psychological health above and beyond personality traits is particularly of interest to military leaders. In this very distinct organization, organizational variables might be even more decisive for employee health than in other organizations, suggesting a pronounced responsibility on the one hand, but also diverse opportunities to strengthening employee health on the other hand. For instance, study satisfaction was related to psychological health both directly as well as indirectly through coping strategies, and showed to be a strong predictor of emotional loneliness and life satisfaction. This knowledge is crucial at the university level and directly implies opportunities for action for enhancing student well-being (e.g., by improving study conditions). Summing up, the military affiliation should be considered at the individual and organization level when working with these samples with the ultimate goal to supporting those efficiently, who are committed to supporting us.

## Data Availability Statement

The datasets presented in this article are not readily available because the data of soldier students is available only under strict restrictions. Requests to access the datasets should be directed to karl-heinz.renner@unibw.de.

## Ethics Statement

The studies involving human participants were reviewed and approved by the Ethik-Kommission der Universität der Bundeswehr München Universität der Bundeswehr München Werner-Heisenberg-Weg 39 85577 Neubiberg. The participants provided their written informed consent to participate in this study.

## Author Contributions

AE and K-HR contributed to the conception and design of the study and organized the data collection. IT and AE performed the statistical analyses. IT wrote the manuscript. All authors contributed to the article and approved the submitted version.

## Funding

The open access publication fees were funded by the University of the Bundeswehr München.

## Author Disclaimer

All statements expressed in this article are the authors’ and do not reflect the official opinions or policies of the authors’ host affiliations or any of the supporting institutions.

## Conflict of Interest

The authors declare that the research was conducted in the absence of any commercial or financial relationships that could be construed as a potential conflict of interest.

## Publisher’s Note

All claims expressed in this article are solely those of the authors and do not necessarily represent those of their affiliated organizations, or those of the publisher, the editors and the reviewers. Any product that may be evaluated in this article, or claim that may be made by its manufacturer, is not guaranteed or endorsed by the publisher.

## Footnotes

^1^Note that Dong et al. (2020) need to be referenced when using the publicly available dataset that can be accessed through https://github.com/CSSEGISandData/COVID-19. This reference introduces the online dashboard in 2020, yet, the dataset itself is updated continuously and thus also holds data from later time points (e.g., as in our case, data from 2021).

^2^In the remainder of the article, we refer to affective organizational commitment when using the terms organizational commitment or commitment.

^3^Although we assessed soldier students’ study satisfaction, we also refer to job satisfaction within this article as soldier students are in a paid employment status which includes the completion of their studies. Therefore, their studies can also be interpreted as jobs.

^4^We additionally tested for mediation effects between the respective predictor at t_1_ and the *change* in the respective outcome variable from t_1_ to t_2_. We could not find any significant indirect effects in these longitudinal mediation models of change.

## Appendix

**Table A1:** Correlations across both measurement points.

Variable	1	2	3	4	5	6	7	8	9	10	11	12	13	14	15	16	17	18	19	20	21	22	23	24	25
1. E _t1_																									
2. E _t2_	.89^***^	---																							
3. N _t1_	−.28^**^	−.26^*^	---																						
4. N _t2_	−.29^*^	−.33^**^	.85^**^	---																					
5. C _t1_	.31^**^	.34^**^	−.32^***^	−.36^**^	---																				
6. C _t2_	.30^*^	.34^**^	−.30^*^	−.41^***^	.91^***^	---																			
7. Com _t1_	.16	.24	−.25^*^	−.12	−.07	−.16	---																		
8. Com _t2_	.12	.19	−.20	−.15	−.11	−.14	.88^***^	---																	
9. StSa _t1_	.30^**^	.26^*^	−.49^***^	−.38^**^	.25^**^	.36^**^	.01	−.02	---																
1. StSa _t2_	.29^*^	.26^*^	−.41^***^	−.41^***^	.39^**^	.43^***^	−.08	−.05	.87^***^	---															
11. AcCo _t1_	.26^**^	.35^**^	.06	.04	.07	−.02	.05	.13	−.01	.07	---														
12. AcCo _t2_	.17	.17	.15	.25^*^	−.35^**^	−.28^*^	.04	.03	−.21	−.16	.46^***^	---													
13. AvCo _t1_	−.13	.05	.52^***^	.30^*^	−.37^***^	−.38^**^	−.18	.02	−.42^***^	−.42^***^	.31^**^	.45^***^	---												
14. AvCo _t2_	.01	−.01	.32^*^	.44^***^	−.51^***^	−.53^***^	.16	.12	−.35^**^	−.43^***^	.24	.61^***^	.63^***^	---											
15. SeSu _t1_	.04	.10	.48^***^	.38^**^	−.08	−.04	−.06	.04	−.32^***^	−.35^**^	.44^***^	.24	.59^***^	.21	---										
16. SeSu _t2_	.02	.02	.28^*^	.42^***^	−.30^*^	−.32^*^	.17	.16	−.32^*^	−.39^**^	.34^**^	.59^***^	.46^***^	.60^***^	.56^***^	---									
17. PoTh _t1_	.17	.11	−.24^*^	−.14	.02	.17	.12	.03	.31^**^	.20	.17	.05	−.07	−.01	−.17	−.09	---								
18. PoTh _t2_	.05	.10	−.13	−.22	.14	.24	−.05	−.02	.18	.16	−.12	.04	.07	−.03	−.08	−.13	.62^***^	---							
19. SoLo _t1_	−.27^**^	−.28^*^	.38^***^	.20	.01	−.03	−.37^***^	−.22	−.24^*^	−.03	−.01	.11	.30^*^	.11	.18	.04	−.17	−.02	---						
2. SoLo _t2_	−.12	−.23	.29^*^	.46^***^	−.11	−.19	−.05	−.07	−.16	−.21	.06	.20	.13	.23	.14	.22	.03	−.19	.58^***^	---					
21. EmLo _t1_	−.09	−.03	.37^***^	.21	−.13	−.17	.03	.19	−.45^***^	−.31^*^	.26^**^	.26^*^	.47^***^	.35^*^	.50^***^	.35^**^	−.22^*^	−.21	.34^***^	.39^**^	---				
22. EmLo _t2_	.04	.03	.26^*^	.35^**^	−.18	−.23	.20	.19	−.43^***^	−.46^***^	.13	.32^*^	.25^*^	.38^*^	.48^***^	.52^***^	−.23	−.23	14	.49^***^	.72^***^	---			
23. LiSa _t1_	.25^*^	.25	−.52^***^	−.41^***^	.19	.30^*^	.26^**^	.07	.55^***^	.46^***^	−.06	−.11	−.53^***^	−.37^**^	−.30^**^	−.21	.20^*^	.14	−.46^***^	−.08	−.47^***^	−.19	---		
24. LiSa _t2_	.34^**^	.33^**^	−.34^**^	−.38^**^	.22	.25	.04	.02	.37^**^	.41^***^	.09	.04	−.31^*^	−.27^*^	−.30^*^	−.24	.41^***^	.28^*^	−.29^*^	−.10	−.35^**^	−.22	.64^***^	---	
25. CoStr _t1_	.06	.22	.42^***^	.22	−.13	−.16	−.10	.25^*^	−.26^**^	−.19	.40^***^	.28^*^	.58^***^	.29^*^	.52^***^	.40^**^	−.17	−.34^**^	.27^**^	.30^*^	.58^***^	.59^***^	−.39^***^	−.23	---
26. CoStr _t2_	.17	.23	.26^*^	.29^*^	−.18	−.23	.21	.12	−.25	−.24	.26^*^	.41^***^	.31^*^	.41^***^	.32^*^	.52^***^	−.32^*^	−.32^**^	.01	.18	.42^***^	.56^***^	−.14	−.13	.69^***^

E, extraversion; N, neuroticism; C, conscientiousness; Com, commitment; StSa, study satisfaction; AcCo, active coping; AvCo, avoidant coping; SeSu, social support; PoTh, positive cognitive restructuring; SoLo, social loneliness; EmLo, emotional loneliness; LiSa, life satisfaction; and CoStr, COVID-19 stress. *N* of sample t_1_ = 106 and *N* of sample t_2_ = 63.

* *p* < .05;

** *p* < .01;

*** *p* < .001.
